# Transcatheter closure of tubular PDA with amplatzer plug 4 in preterm infants weighing between 900 and 3,400 g: the pros and cons

**DOI:** 10.3389/fcvm.2023.1283992

**Published:** 2023-10-13

**Authors:** Nathalie Mini, Martin B. E. Schneider, Katja Schneider

**Affiliations:** ^1^ Department of Cardiology, German Paediatric Heart Centre, University Hospital of Bonn, Bonn, Germany; ^2^ Department of Neonatology, GFO Kliniken, Bonn, Germany

**Keywords:** tubular duct, transcatheter PDA closure, preterm, plug 4, PDA

## Abstract

**Objective:**

To our knowledge, no prior study has focused on the outcome of PDA occlusion using an Amplatzer™ Vascular Plug 4 (AP4) in ill preterm infants. This study aims to highlight the pros and cons of AP4 in this cohort.

**Methods:**

Between 2020 and 2022, 26 ill preterm infants underwent PDA closure in our centre. The median age, weight, and gestational age were 60 days (11–180 days), 1,900g (900–3,400 g), and 25 weeks (22–33 weeks), respectively. The indication of the intervention was hemodynamically significant PDA. A medical trial with non-steroid medication failed to close the ducts in all patients. Follow-up using echocardiography was done 24, 48, and 72 h after the intervention.

**Results:**

Of 26 ducts, 21 were successfully closed with AP4. Five ducts shorter than 7 mm were unsuitable for AP4 and were closed with the Amplatzer Piccolo device. The median radiation time was 4 min (3–9 min). No early plug-related complications or deaths were documented. Plug-related jailing of the left pulmonary artery as a late complication was 9.5%, and LPA reintervention was required. All ducts were closed after 48 h.

**Conclusion:**

Implantation of the AP4 using a 4 F 0.38 guide wire-compatible catheter without inserting a long sheath makes the closure of tubular ducts with this device feasible and uncomplicated with a short intervention time. However, the limited sizes with fixed lengths of the AP4 make it unsuitable for ducts wider than 4.5 mm and shorter than the chosen device length, which can increase the risk of significant left pulmonary stenosis. A wide range of plug diameters and lengths is required to accommodate the large and short ducts.

## Introduction

A patent ductus arteriosus (PDA) is the most frequent pathophysiological problem in preterm infants and is associated with several adverse clinical conditions. The persistence of the ductus is inversely proportional to the gestational week. The established primary therapy is the administration of non-steroidal anti-inflammatory drugs; however, especially in extremely premature infants, the therapy is occasionally unsuccessful, or the side effects require the termination of the drug therapy (1–3).

Transcatheter closures are safe and feasible in very low-weight infants and have become an alternative to surgical closure (4). Several vessel occluder devices have been used to close the ducts in preterm babies (5–8). Amplatzer Plugs, including II and 4 and the Micro Medtronic Plugs, have shown promising results in closing the tubular ducts (9).

Moreover, the Amplatzer Piccolo Device has had excellent results in closing the short ducts.

Some ducts in preterms and those with a history of preterm birth have particular properties for enlargement after being crossed or after implanting the device. In this report, we aim to review our centre's results in closing the tubular ducts with AP4 in preterms and to highlight its pros and cons.

## Patients and methods

Between 2020 and 2022, 26 preterm infants who underwent trans-catheter PDA closure were retrospectively recruited. All infants were hospitalized and not discharged since birth due to prematurity-related problems (Table 1). The indication for PDA closure was pulmonary over-circulation in 8 and severe bronchopulmonary dysplasia (BPD) with its related respiratory problems in 18 patients. A medical trial with non-steroid medication had failed to close the ducts in all these patients. Two patients were intubated during the intervention. Based on the Krichenko classification (10), the duct was Type B in 4, Type E in 9, and Type C in 13 patients. The following points were documented: the shape of ducts, early and late complications, radiation time, the need for a ductal size-related device change, failed transcatheter closure, and time of ductal closure. The diameter and length of the duct for choosing the device size were measured by angiography (lateral view). Echocardiography was used for follow-up and was done 24, 48, and 72 h after the intervention.

**Table 1:** Summary of patients’ characteristics and the types of ducts.

Pat	GA	BW	Age	Weight	HI	BPD	Type	Device	IVH	CPAP
1	29	440	85	1,300	Yes		E	AP4	Yes	
2	25	745	19	900	Yes		B	Piccolo		
3	25	745	20	900			B	Piccolo		
4	25	750	22	1,600			B	Piccolo	Yes	
5	31	1,000	11	1,200	Yes		C	Piccolo		
6	26	735	60	1,800	Yes		E	AP4		
7	23	480	26	1,300		Yes	E	AP4		Yes
8	28	1,370	65	3,000			C	AP4	Yes	
9	24	785	70	2,700	Yes	Yes	C	Ap4		
10	25	320	180	2,900	Yes	Yes	C	AP4	Yes	Yes
11	26	480	60	2,600	Yes	Yes	C	Ap4	Yes	
12	24	590	60	2,600		Yes	C	AP4		
13	22	481	150	3,000	Yes	Yes	C	AP4	Yes	Yes
14	28	860	76	2,600		Yes	C	AP4		Yes
15	27	790	21	900	Yes	Yes	C	AP4	Yes	
16	24	470	120	1,800	Yes	Yes	C	AP4	Yes	Yes
17	24	470	60	1,800	Yes	Yes	C	AP4		
18	23	955	75	2,800	Yes	Yes	C	AP4	Yes	Yes
19	34	1,900	45	2,000	Yes	Yes	E	AP4		Yes
20	27	540	60	2,300		Yes	E	AP4		Yes
21	26	940	60	1,800		Yes	E	AP4		
22	30	1,600	30	2,300	Yes	Yes	E	AP4		
23	29	450	90	1,000	Yes	Yes	C	AP4		Yes
24	23	450	120	3,000	Yes	Yes	E	AP4		Yes
25	22	450	150	3,400	Yes	Yes	E	AP4	Yes	Yes
26	24	705	45	1,200	Yes	Yes	C	Piccolo	Yes	

GA, gestational age (in weeks). HI, heart insufficiency; BPD, bronchopulmonary dysplasia; BW, birth weight in grams; IVH, intraventricular haemorrhage; CPAP, continuous positive airway pressure.

### Statistical analysis

All statistical analyses were performed using SPSS version 22. Continuous variables were reported as median ± standard deviation (SD), and categorical variables as count (percentage).

### Ethical statement

The study complies with the Declaration of Helsinki (as revised in 2013). Owing to the purely retrospective study design, using available institutional clinical records, with an absence of impact on the management of the patients included and completely anonymous data presentation, ethical approval was not necessary. Parental consent for using the device was obtained before the interventions in all patients.

### Device description

The AP4 device (Amplatzer™ Vascular Plug 4) is made from nitinol and attached to a 155-cm-long PTFE-coated delivery wire in a hoop dispenser. The device is loaded in a short introducer and is compatible with a 0.038-inch diagnostic catheter. A plastic vice is included and may be attached to the delivery wire to facilitate the device's detachment by rotating the delivery wire anticlockwise until it separates from the device.

**Figure 1:**
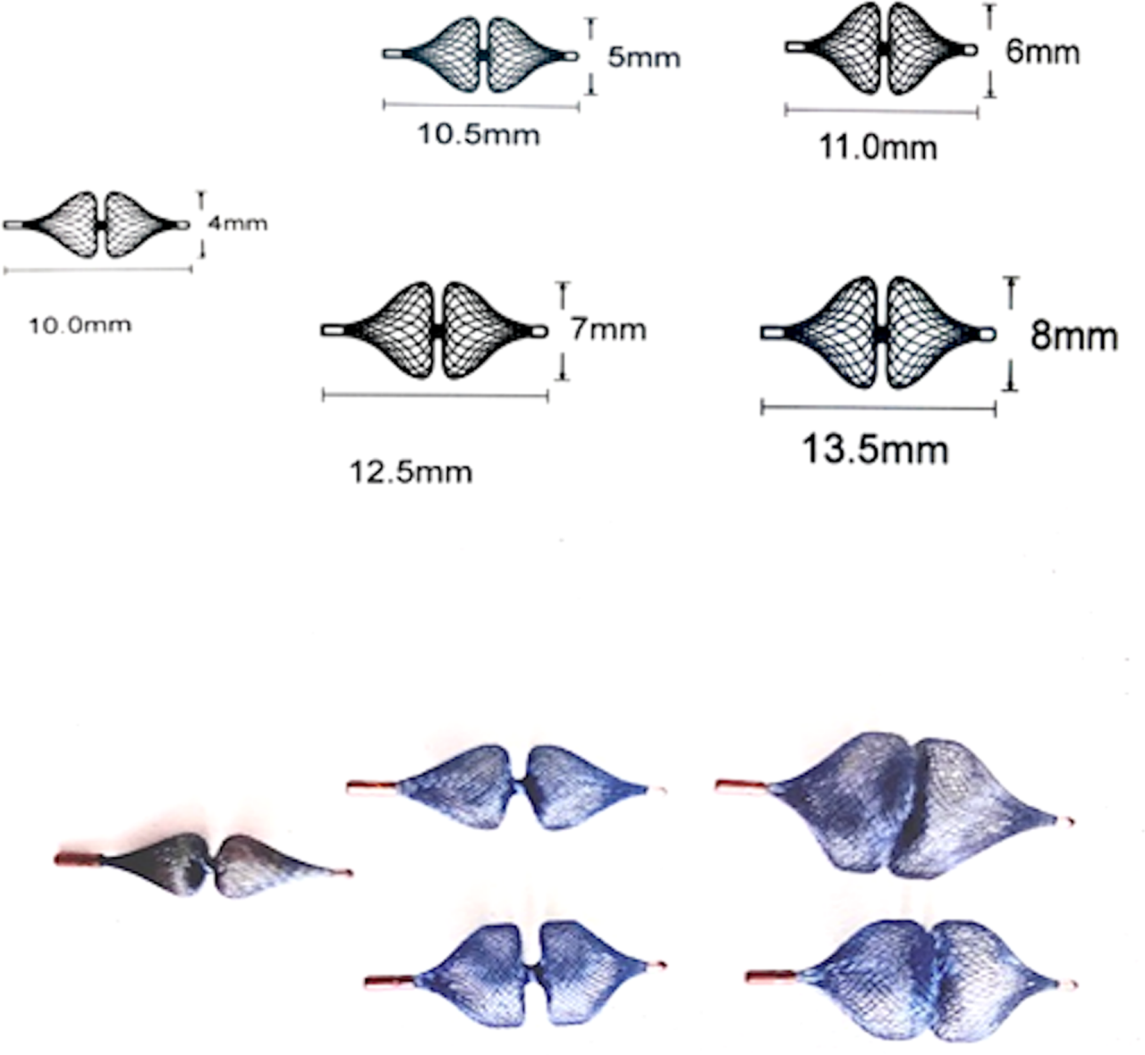
Demonstration of the available sizes of the amplatzer plug 4 with their related lengths. The device is available in the following sizes (diameter/length): 4/10 mm, 5/10.5 mm, 6/11 mm, 7/12.5 mm, and 8/13.5 mm.

The following sizes of the AP4 are available: Plug 4.4/10 mm, Plug 4.5/10.5 mm, Plug 4.6/11 mm, Plug 4.7/12.5 mm, and Plug 4.8/13.5 mm (Figure 1). The device is used for embolization of major aortopulmonary collateral arteries (MAPCAs), fistulas, paravalvular leak and PDA closure. The results showed that the device is safe and effective for PDA closure; however, unlike the Amplatzer Piccolo device, the AP4 has no Food and Drug Administration (FDA) approval for closure of PDA in premature infants.

### Procedure description

All interventions were done after getting parental consent to use different devices, including the AP4. No heparin bolus was given before or during the intervention to accelerate the possession of PDA closure.

Twenty-four interventions were done under sedation, and 11 were under continuous positive airway pressure (CPAP). Two intubations were required for severe BPD. In preterm patients with a weight under 3 kg, the femoral vein was accessed using a 4 F sheath (Terumeo, Radifocus® introducer II). Under fluoroscopy, the tricuspid valve and pulmonary valve were accessed using a 4F Gleidcath (Terumo®, Radfocus®, Gleidcath™) or a 4F Judkins's suitable catheter (Cordis®, JR, 4F), and the duct was crossed using GLIDEWIRE® Hydrophilic Coated Guidewire or WIZDOM™ Steerable Guidewire - Cordis.

The duct size was evaluated with either a contrast agent or echocardiography in the case of echocardiogram-guided closure. The device was chosen to be 50% larger in diameter than the duct diameter (Table 2). A 0.35 wire (Fixed Core Wire Guide straight) was positioned in the descending aorta. Unlike the 0.38 guidewire-compatible diagnostic catheter, in which the AP4 will be directly employed as a loading catheter, the device cannot be delivered through a Gleidcath because its 0.35 lumens do not accommodate the device, and replacement of the accommodated catheter is required.

**Table 2:** Duct measurements and the used AP4.

PDA	Duct diameter (mm)	Duct length (mm)	Tool used for measurement (angiography or echo?)	Size of AP4 (mm)	Length of AP4 (mm)	AP4 compressed by the duct with no protrusion in PA or DAO?
1	4	11	Echo	8	13.5	Yes
2	3	10	Angiography	6	11	Yes
3	2.5	8	Angiography	5	10.5	Yes
4	2.5	7	Angiography	5	10.5	Yes
5	2.5	10	Angiography	5	10.5	Yes
6	4	10	Angiography	8	13.5	Yes
7	4	11	Angiography	8	13.5	Yes
8	3	8	Angiography	6	11	Yes
9	3.5	8	Angiography	7	12.5	Yes
10	2.5	8	Angiography	5	10.5	Yes
11	4	8	Angiography	8	13.5	No
12	2.5	8	Angiography	5	10.5	Yes
13	3.5	11	Angiography	8	13.5	Yes
14	2.3	8	Angiography	4	10	Yes
15	3.7	10	Angiography	8	13.5	Yes
16	4.5	9	Angiography	8	13.5	Yes
17	3.5	7	Angiography	7	12.5	No
18	4	10	Echo	8	13.5	Yes
19	2.5	10	Angiography	5	10.5	Yes
20	2.5	9	Angiography	5	10.5	Yes
21	2	8	Echo	4	10	Yes

AP4, amplatzer 4; PA, pulmonary arteries; DAO, descending aorta.

The wire was removed after advancing a 0.038 guidewire-compatible diagnostic catheter and positioning it in the descending aorta. The chosen plug was thoroughly flushed with heparinized saline through the stop cock, and the tip of the loader was tapered into the hub of the catheter. The rotating luer was pressed into the hub of the catheter and rotated clockwise to ensure full engagement of the loader to the catheter hub. Holding the catheter with the left hand, the delivery wire of the device was then slowly advanced into the catheter until the double marks of the device were seen at the tip of the catheter in the descending aorta. The delivery wire was held with the right hand and was, together with the delivery catheter, retracted to the aortic isthmus region. The catheter was then retracted alone till the distal part of the plug was deployed. The catheter and the delivery wire were retracted until the distal part was in its ductal position, then the catheter was retracted till the pulmonary part of the plug was deployed. Usually, the trachea will be a golden landmark for deploying the pulmonary part of the plug.

**Figure 2:**
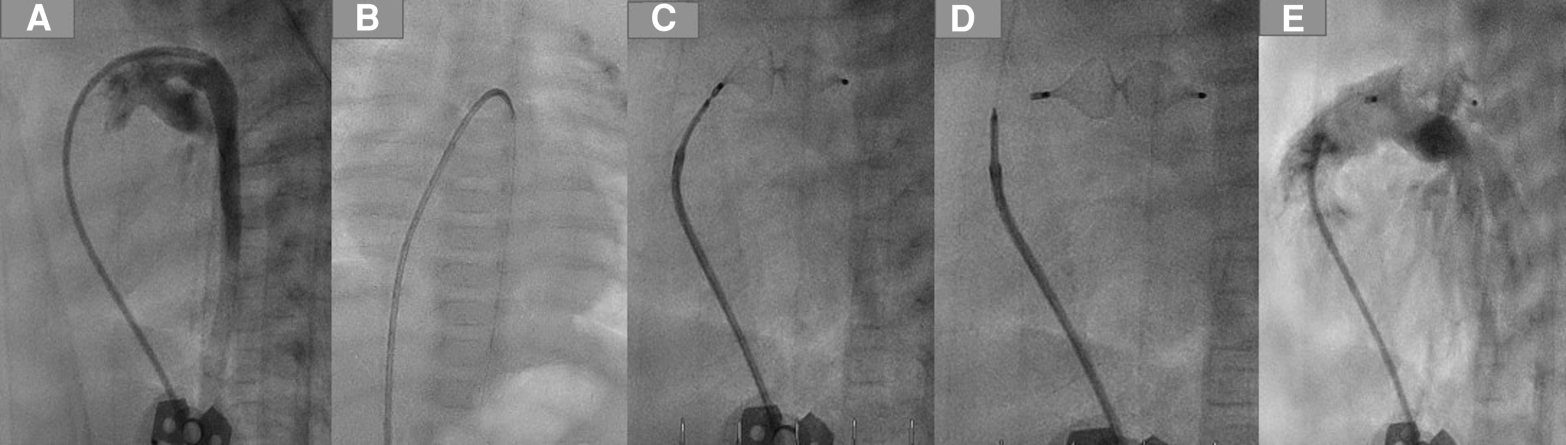
PDA closure with AP4 step by step. (**A**) Demonstrating the duct using a 4 F JR, which crossed the duct and was positioned in the descending aorta. (**B**) A 0.14 coronary wire was positionedin the DAO. (**C**) Plug 8/13.5 was employed in the duct. (**D**) Removing the delivery wire from the plug. (**E**) Angiography after the intervention, no residual shunt.

After deploying the device, we performed echocardiography to ensure the device's position and exclude a residual shunt and any device-related pulmonary stenosis or aortic stenosis. After verifying the device's position and the intervention's results, the delivery cable was detached from the device by rotating it anticlockwise using the plastic vice until it was wholly separated from the plug. The cable with the catheter and the short sheath were removed (Figure 2).

For some children weighing more than 2,500 g (*n* = 9), the femoral artery was accessed using a 3.2 F sheath to perform angiography in the descending aorta to evaluate the results of the intervention.

## Results

The median age, weight, and gestational age (GA) at the intervention time were 60 days, 1,900g, and 25 weeks, respectively. The intervention was successful in all preterm infants. In two ducts, more than one attempt with more than one device was required to adapt to the large duct size. The Amplatzer Piccolo device was considered as the first option to close a short duct in 5 patients.

### Trans-catheter PDA closure with AP4

The intervention was done under sedation in 19 patients, 9 with CPAP. Two babies were intubated due to severe BPD. The ducts were closed using AP4 in 21 preterm babies. All interventions were successful, and an echocardiogram-guided closure was done in 3. The median radiation time was 4 min (3–9 min).

In two infants, the AP4 had to be replaced with a larger one to adapt to the ductal enlargement after crossing or after the first attempt to close it. The device was easily retracted in the delivery catheter. There was no plug-related complication during the intervention or plug-related death. In all babies with pulmonary hypertension, we observed a short time change of the ST segment in ECG while crossing the tricuspid with a secondary reduction in blood pressure, and the need for volume substitution and low short-term doses of catecholamine were required in 3 infants.

A small residual shunt at the end of the intervention was documented in all patients.

There was no device embolization, no plug-related haemolysis or infection, and no tricuspid or pulmonary damage.

### Follow-up and late complications

In follow-up, the ducts were closed in all infants 48 h after the intervention without a residual shunt. There was no tricuspid damage or worsening of the tricuspid or pulmonary regurgitation, no aortic stenosis, and no significant left pulmonary artery (LPA) stenosis.

**Figure 3:**
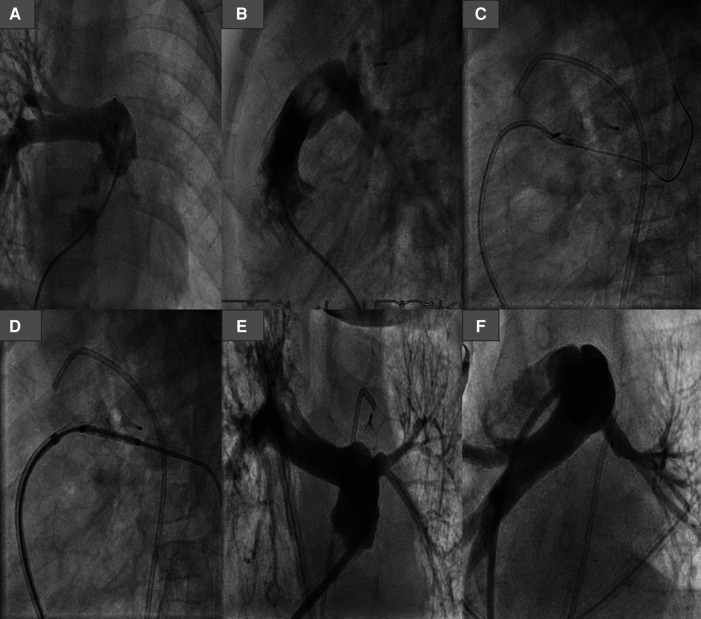
Plug-related left pulmonary obstruction with the required reintervention. (**A,B**) LPA obstruction in AP and lateral view. (**C**) Crossing the LPA using a coronary wire. (**D**) Balloon premounted stent positioned in LPA. (**E,D**) LPA after stenting.

An AP4-related left pulmonary stenosis was documented in 2 patients born at 31 and 24 weeks' gestation and weighing at the intervention time 2,600 and 2,500 g, respectively. The first patient's duct was Type C and was occluded with an AP4 8 × 13.5 mm. The native LPA was hypoplastic (2 mm) and jailed by the AP4, causing LPA obstruction 3 months after the intervention. After discussing the surgical removal of the plug with PA plastic vs. reintervention by the surgeons, the decision for reintervention was made for the following reasons: first, the native LPA was hypoplastic (<2 mm), and the obstruction of the LPA was extended to the Helios; second, the patient had severe BPD and pulmonary hypertension (PH), which can increase the risk of the surgery. For these reasons, reintervention was in this patient superior to surgery. A rash reintervention was performed to recanalize the obstructed LPA; the balloon dilation of the recanalized LPA was ineffective, and a stent was required and implanted. Echocardiography in follow-up showed satisfactory development of the LPA (Figure 3). In the second patient, the LPA (3.5 mm) was jailed by the pulmonary side of the AP4 (7 × 12.5 mm), causing a narrowing of the LPA entrance (2 mm) without restriction of the LPA perfusion. We dilated the LPA 4 months after the closure of the duct. In follow-up, there was no need for a reintervention.

Two days after the intervention, a thrombus in the right atrium was documented in 1 patient and was treated with low-molecular-weight heparin (Clexane®).

Four patients with severe bronchopulmonary dysplasia died 3 and 4 months after the intervention due to prematurity-related causes.

## Discussion

Whether duct closure in ill preterm infants could lead to better outcomes by reducing morbidity and mortality is controversial and still needs a consensus among authors. However, some studies have shown that some patients are likely to benefit from duct closure, such as preterm infants less than or equal to 26 weeks GA or those with severe hemodynamic disturbances (11).

Several devices are frequently used for duct closure in preterm infants: the Amplatzer Ductus Occluder II (ADO II AS), Piccolo Occluder Device from Abbott, Amplatzer Plug II, and Medtronic Microvascular Plugs. Using the Amplatzer Vascular Plug 4 to close hemodynamically significant ducts was reported in a case report that included 1 preterm patient weighing 2,100 g (12) and in infants weighing more than 2,500 g (13) and more than 4,100 g (8).

In this study, we focus on using AP4 to close the ducts in very ill preterm babies who were hospitalized since birth due to prematurity-related problems and weighed between 900 and 3,400 g.

The duct closure using AP4 in this particular cohort is feasible and straightforward.

Using a 4 French, 0.38 guidewire-compatible diagnostic catheter without a long sheath, the intervention is performed more quickly than with other devices. It also spares several manoeuvres in the region of the tricuspid and pulmonary valves, reducing the related possible undesired trauma. Introducing the device via 4 French sheaths widens the access possibilities for the duct closure and accommodates the vessels' size in very small babies. The device has excellent echocardiographic visibility, making echocardiogram-guided intervention an alternative to fluoroscopy. The low profile of the AP4 allows the easy retraction of the device in the delivery catheter in the case of repositioning or replacement with another device. The two marks on the plug edges are excellent markers that help to recognize the plug position for snaring in the case of embolization.

However, the available lengths of Plug 4 make it suitable only for tubular ducts. In the case of short ducts, the plug may protrude into the isthmus region or the pulmonary artery, leading to significant stenosis. Ducts with a diameter of more than 4 mm may be amenable to being closed with the plug because of the limited device size (4–8 mm). In some cases of tubular ducts, the plug's implantation could be inconvenient due to the fixed plug size with fixed lengths—the greater the desired diameter, the longer the device. Based on our results, the jailing of the LPA with the need for reintervention was observed in 9.5% of patients (4.25% LPA obstruction and 4.25% mild stenosis). Two long plugs were used in these infants, 12.5 mm and 13.5 mm, to close 3.5- and 4-mm tubular ducts (with duct lengths of 7 and 8 mm respectively). In these cases, the two devices were longer than the ducts, causing a significant pulmonary stenosis with obstruction in one of them and a mild stenosis in the second one. In the patient with LPA obstruction, the native LPA was hypoplastic (<2 mm), and the narrowest part of the PDA was at the pulmonary site, leading to the pulmonary site of the plug protruding into the PA, causing LPA stenosis, which progressed to LPA obstruction detected 3 months after the intervention. The incidence of LPA obstruction in our cohort was higher than in other studies (9, 14, 15), raising concerns about using the AP4 in the case of hypoplastic LPA (4.24% of the patients) due to an increased risk of device-related significant LPA stenosis and obstruction.

In other studies using different devices to close the PDA in preterm infants (Amplatzer Plug II, Amplatzer Piccolo device), severe device-related LPA stenosis with or without the need for stenting was observed (9, 14–16), especially when the LPA was less than 3 mm (14); this observation is in agreement with our results, with increased incidence of mild and severe device-related LPA stenosis in our cohort due to the length of the device compared with the length of the occluded ducts.

In the two previous studies that used a Piccolo occluder to close the PDA (7, 17), occluder-related LPA stenosis was not documented in the first study (7). It occurred in 3 patients (0.5%) in the second report (17). However, device-related significant aortic stenosis was reported in two studies, with an aortic obstruction and need for stenting in one patient (7, 16); this observation was in contrast with our results, in which there was no AP4-related aortic stenosis. The embolization of the device was 2% after PDA closure using a Piccolo device (17), compared with no embolization in our cohort with AP4. In contrast to the previous study, in which 5 patients ≤2,000 g had worsening tricuspid regurgitation after the intervention and one patient had a device-related haemolysis (7), no AP4-related tricuspid damage or haemolysis happened. Piccolo-related cardiac perforation with death was reported (17), compared with no AP4-related death or perforation.

In 2015, Philip et al. (18) described a new type of PDA in preterm infants (Type F), which has a larger diameter and is longer than Types A-E. Although there was no Type F in our cohort, its length and shape seem appropriate to be closed with AP4. At the same time, the fact that Type F tends to have a large diameter makes the AP4, with its limited available sizes of 4–8 mm, not always appropriate for closing such ducts, and PDA closure with Plug II could be superior to AP4, especially when the duct diameter is more than 4 mm.

## Conclusion

Transcatheter occlusion of the tubular ducts using AP4 in ill preterm infants weighing between 900 and 3,400 g is feasible and uncomplicated. Using a 4 French, 0.38 guide wire-compatible catheter as a delivery catheter without a long sheath makes the closure of the duct in this cohort easier, with a relatively short intervention time, and could spare the patients undesired worsening of tricuspid and pulmonary regurgitation. However, the limited sizes of the plugs were unsuitable for ducts wider than 5 mm or shorter than 7 mm. The larger the device's diameter and the longer its length, the higher the risk of plug-related jailing of the LPA, making the intervention inconvenient for the tubular ducts shorter than the chosen device and in the case of hypoplastic LPA, which increases the risk of LPA obstruction. A wide range of plug diameters and lengths is needed to accommodate the ducts wider than 4.5 mm and shorter than 7 to 8 mm.

## Data Availability

The original contributions presented in the study are included in the article/**Supplementary Material**, further inquiries can be directed to the corresponding author.
